# First Data in the Process of Validating a Tool to Evaluate Knowledge, Attitude, and Practice of Healthcare Providers in Oral Care of Institutionalized Elderly Residents: Content Validity, Reliability and Pilot Study

**DOI:** 10.3390/ijerph18084145

**Published:** 2021-04-14

**Authors:** Florence M. F. Wong

**Affiliations:** School of Nursing, Tung Wah College, Hong Kong, China; florencewong@twc.edu.hk; Tel.: +852-3468-6838

**Keywords:** elderly, oral care, healthcare providers, knowledge, attitudes, practices, tool, validation

## Abstract

Background: Oral health of elderly people is a global concern. Poor oral health in institutionalized elderly people has been attributed to poor knowledge, attitude, and practice (KAP) of healthcare providers. However, no validated KAP tool is available yet. Objective: To develop and validate a tool to measure knowledge, attitude, and practice of healthcare providers in oral care of institutionalized elderly people. Methods: The development and validation of the tool was based on literature reviews, comments from professional experts, and statistical analytic methods. Content validity in the instrument psychometric property and its relevance with reliability are essential. Content validity ratio and content validity index were performed. Then, a pilot study was conducted in 20 institutionalized healthcare providers for testing applicability, feasibility, and reliability. Results: A total of 43 items were developed in three domains, knowledge (19 items), attitude (13 items), and practice (11 items). Content validity analysis revealed the KAP tool with high values of the I-CVI (score 1.00) and S-CVI (S-CVI/UA result 1.00). The test-retest reliability with Cronbach’s alphas of knowledge, attitude, practice, and overall KAP were 0.67, 0.93, 0.92, and 0.94, respectively. Conclusions: The developed and validated tool is appropriate to measure KAP of healthcare providers in oral care of institutionalized elderly people. It can be used to measure KAP of institutionalized healthcare providers in order to develop appropriate strategies to improve KAP of healthcare providers.

## 1. Introduction

### 1.1. Background

Oral health in elderly has raised global concern [1]. Poor oral health is closely linked to deteriorated general health conditions that increase morbidity and mortality. Consequently, the burden on healthcare services is increased [1]. Many oral problems cause eating problems, contributing to weight loss and nutritional deficiencies. Apart from physical vulnerabilities, the psychosocial aspect is also affected as dental problems increase communication difficulty and social isolation due to low self-esteem [1]. A study conducted in Hong Kong reported multiple oral problems in elderly people from aged 65 to 74, including dental caries, root caries, gum bleeding, and deep pockets [2]. It indicated that maintaining good oral hygiene and early dental treatment were inadequate among the elderly. Many elderly people used toothpicks instead of dental floss [3]. They also did not habitually brush their teeth twice a day with toothpaste, or/and rinse their mouth [4]. Due to inadequate support and lack of insurance for dental consultation, elderly people tend to ignore regular dental checkups [5]. In addition, most of the elderly people believed that it was normal to lose their natural teeth when getting old and that they could use dentures to compensate the tooth loss [5]. In general, elderly people are more likely to have long-term illnesses and they may need to take multiple medications, which induce hyposalivation. Their oral conditions may further deteriorate. Other factors, such as degrees of cognitive function and self-care independence, can affect oral care practice among elderly people [6,7,8]. More importantly, those who had poor oral health manifested other functional problems, such as eating and chewing problems, speech difficulty, and malnutrition [9]. Elderly people with cognitive problems may forget about oral care. Those with physical restrictions will exhibit self-care dependence. As a result, their oral care may be compromised [10,11,12]. Eventually, their overall general health and quality of life will be affected [13]. Therefore, oral health evaluation has been one of the most important health assessments, particularly in elderly people. Oral health of elderly people has been commonly assessed by validated instrument such as the Geriatric Oral Health Assessment Index (GOHAI) questionnaire to evaluate the subjective perception of an elderly’s oral health condition [14]. Like another instrument, the National Health and Disability Survey, it is to evaluate the use of care and the oral health status of elderly people aged 60 years or older [15].

Considering elderly people in long-term care (LTC) institutions, their oral health is found relatively poorer than those in the community [11,13]. In Hong Kong, most elderly people with limited self-care ability due to physical and mental disabilities are arranged to long-term care institutions for continuous care [4,5]. Their daily oral care may be dependent on institutionalized healthcare providers. However, more severe oral problems have been identified, such as broken teeth, cracked and sore lips and mouth, ill-fitting dentures, bleeding gum, and toothache [5]. In the Chinese population, most elderly people only visit the dentist when they have oral problems [11]. Institutionalized elderly may have more difficulty in having regular dental visit [5]. A systematic review on oral health-related quality of life and associated factors among institutionalized elderly people showed that institutionalized elderly people had relatively poor oral health with multiple dental and periodontal problems. Factors associated with oral health and oral health-related quality of life were identified and categorized into non-modifiable and modifiable factors. Non-modifiable factors were age, gender, and educational level. Modifiable factors included low dental service accessibility, limited self-care ability, and socioeconomic factors, such as inadequate health insurance coverage, difficulty in attending dentist, or existence of clinical treatment [16]. More importantly, maintenance of oral health by, for example, regular and necessary dental checkup and treatment in institutionalized elderly is more dependent on healthcare providers. However, poor oral health of institutionalized elderly people has been documented and closely attributed to poor knowledge, attitude, and practice (KAP) of healthcare providers [17,18,19].

Oral care to elderly residents is a basic care routine of healthcare providers. Inadequate knowledge of oral care is evidenced as a key factor to deprioritize and undermine oral care [13,17]; subsequently, attitude and practice of oral care are affected. KAP are interrelated and commonly examined to understand specific constructs in various studies [19]. Studies investigating KAP toward oral care increased in these two decades, but in other regions [13,19,20,21]. Their instruments were developed in other languages and based on the authors’ professional experiences [19,22,23]. Some studies did not report reliability results of the instruments [13,19,20,21]. Besides, those studies lacked a health-related model to guide the development of KAP. To better understand KAP of healthcare providers in oral care for elderly people in the LTC institutions, this study used the capability, opportunity, motivation, and behavior (COM-B) model to guide the develop the tool and to illustrate relationships of KAP of healthcare providers in oral care of institutionalized elderly people [24]. Capability embraces knowledge and skills. Opportunity implies possible factors that make specific behavioral changes. Motivation is related to decision-making with action through a process of emotional and analytic responses. At last, health behavior is determined by and contributed to certain practice. Based on this model, oral care ability is determined by the level of knowledge and skills of healthcare providers. Factors influencing oral care practice, such as personal experience or attitude toward oral care of elderly residents and institutionalized policy or regulation, should be considered. It is decisive to the practice of healthcare providers who are responsible for performing oral care for elderly residents. Therefore, the constructs (KAP) of the tool were developed.

### 1.2. Operational Definition

- **Knowledge:** Knowledge reflects how a healthcare provider understands the concept of oral health, oral problems, and related causes and symptoms, about oral care and its importance in oral health and general health considering oral and physical conditions of an elderly resident [10,19].
- **Attitude:** Attitude is defined as a learned predisposition to think, feel, and act of a healthcare provider in a particular way towards an elderly resident who needs oral care. Attitude also reflects how the healthcare provider values oral care for an elderly resident [12,22].
- **Practice:** The individual involvement in performing oral care as a preventive measure to maintain oral health of an elderly resident. Practice also reflects a healthcare provider’s behavior or reaction that should be done in a situation of the elderly resident [22,23].

### 1.3. Aims

This study’s aim was to develop a local KAP tool to measure KAP of healthcare providers in oral care for institutionalized elderly residents. The healthcare providers include healthcare workers, healthcare assistants, and nurses, whose one of the major duties was to perform oral care for elderly residents.

## 2. Materials and Methods 

### 2.1. Design

This study was divided into three parts, development of initial KAP tool through literature review and consultation of experts, content validity index to ensure item applicability and appropriateness, and a pilot study for test-retest reliability and feasibility of the tool [21,25,26,27]. 

### 2.2. Literature Review

The search was limited to studies (1) published between years January 2011 and October 2020; (2) which are primary studies that examined knowledge, attitude, or practice of healthcare providers in oral care, (3) in which participants were elderly, (4) with abstracts available, and (5) which were written in English or Chinese through the electronic databases. Studies published in 10 years were included because they were recent and were able to more reliably reflect the current practice since the technology and needs of healthcare services have been changing quickly. Primary studies examined KAP directly. Their results were more valid and representable. Articles in Chinese or English were included to increase the search spectrum and obtained more relevant evidence. However, the exclusion criteria were clinical guidelines or recommendations, editorials and reports of expert opinions. 

The comprehensive search was done by a research assistant. He/she was taught to search for relevant studies using the available databases, including PubMed, MEDLINE (OvidSP), EMBASE, and CINAHL. The relevant articles were identified using the keywords in the title, abstract, or subject descriptor/MeSH terms. All included studies that met the inclusion criteria were retrieved. Chinese studies utilizing Chinese keywords were also searched. Lastly, Google Scholar and a hand search of reference list of the relevant studies based on the study title were used. The keywords were ‘knowledge’, ‘attitude’, ‘practice’, ‘oral care’, ‘oral health’, ‘elderly’, ‘residents’, ‘long-term care institution’.

**Figure 1:**
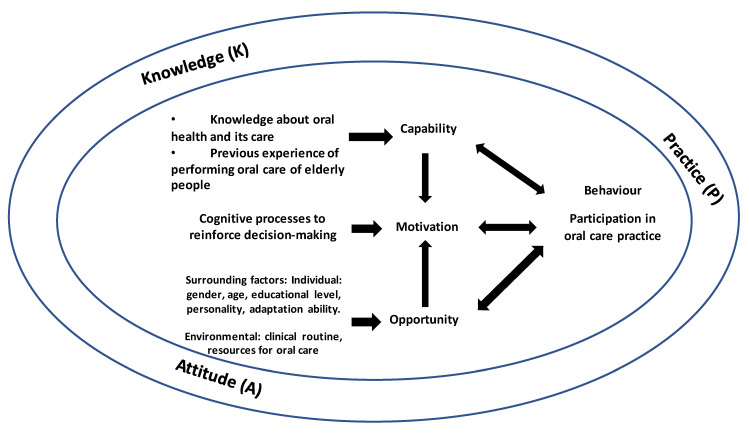
The Capability, Opportunity, Motivation Behavior (COM-B) Model and knowledge, attitude, and practice.

As many items as possible were mainly selected and developed according to the appropriateness of the past relevant studies. The COM-B model was used to guide the domain identification. Figure 1 illustrates the COM-B model and KAP.

### 2.3. Consultation from Experts in Two Stages 

There were two stages of expert consultations. Different experts were involved in these two stages for ensuring the items were more relevant and appropriate to meet the requirement of understanding KAP of healthcare providers in oral care of institutionalized elderly people. 

At the first stage, three experts (a physician specialized in oral health in elderly and two dentists in community clinics) were invited to give their comments on the drafts of the KAP tool (English and Chinese version) independently. Their roles were to evaluate the overall format, domains, rating method, and items of the tool independently. The draft was modified according to the comments from these three experts. 

At the second stage, another three experts (one community dentist, one dentist psychologist, and one dentist in faculty of dentistry of a university) were invited to evaluate the tool independently [20]. They evaluated the domains and items for their appropriateness, structure and clarity, redundant inquiries, and ambiguity of meaning. Modification or elimination was made if necessary. 

### 2.4. Tool Translation and Interviews

Based on the literature review, an initial draft of KAP questionnaire was then developed. The English version was translated to Chinese. Back-translation was reviewed by a technical expert fluent both in English and Chinese. The two versions were finalized when the experts in the first stage were satisfied with them. Then, the tool was developed for content validity index (CVI). Interviews were conducted to examine how respondents understand, interpret, and answer each item [21]. Modification or rephrasing was made if needed.

### 2.5. Content Validity

Content validity index (CVI) reports the content validity in tool development using item-CVI (I-CVI) and scale-CVI (S-CVI) [25,26]. The I-CVI is calculated as the number of experts rating each item from a scale of ‘very relevant’ (score 1), ‘relevant’ (score 1), ‘irrelevant’ (score 0), and ‘very irrelevant’ (score 0) divided by the total number of experts. The value range of I-CVI >0.79 indicates the relevant item, between 0.7 and 0.79 item for revision, and below 0.7 item for elimination. The universal agreement (UA) among experts (S-CVI/UA) was calculated by the sum of all items with I-CVI equal to 1 divided by the total number of items for the S-CVI [26]. A S-CVI/UA ≥0.8 indicates excellent content validity [27]. To obtain CVI for relevancy and clarification of each item, five experts, including dentists in university dentistry, dentists in community, dental hygienists and dental nurses, were recruited for judging the items using the rating from 1 (not very relevant) to 4 (very relevant). Items were rephrased when they were rated lower than 3 with comments. Content validity ratio (CVR) is another empirical analysis to measure the essentiality of an item [25]. The formula for the CVR is CVR = (Ne − N/2)/(N/2). Ne is the number of experts indicating an item as ‘essential’ and N is the total number of experts [26].

### 2.6. A Pilot Study

A pilot study was conducted to test the feasibility and practicality of the study. A total of 20 subjects, who are healthcare providers responsible for oral care for elderly in LTC institutions, were recruited. A test-retest reliability was performed, indicating that the subjects would be required to fill in the KAP questionnaire on the first time and about 10 to 14 days later [26]. 

The study was commenced after ethics approval was obtained. Two LTC institutions were contacted for subject recruitment and data collection. The logistics were discussed with the site managers. As for data collection, an online consent following information sheet was needed after the subjects understood the study purpose and procedure. The data were collected via online Google survey. At first, subjects were required to complete the demographic form and KAP questionnaire; after 10 to 14 days, they were requested to complete the KAP questionnaire only. All data were kept confidential, encrypted, and stored. After data analysis, all information with personal particulars was permanently deleted. The data were only accessed by the research team. All data were stored for five years before permanent deletion.

### 2.7. Ethical Considerations

An approval was sought from the research ethics committee of the study educational institution prior to commencement of the study. Implied consent was taken when participants agreed to complete the questionnaires. They were assured that all data related to their personal information would be kept strictly confidential. Data were collected via face-to-face interviews, telephone interviews, or online Google questionnaire, from 31 October 2020 to 11 January 2021. The completion of questionnaires took about 20 min.

## 3. Results

### 3.1. Content and Domain Specification and Item Generation

**Figure 2:**
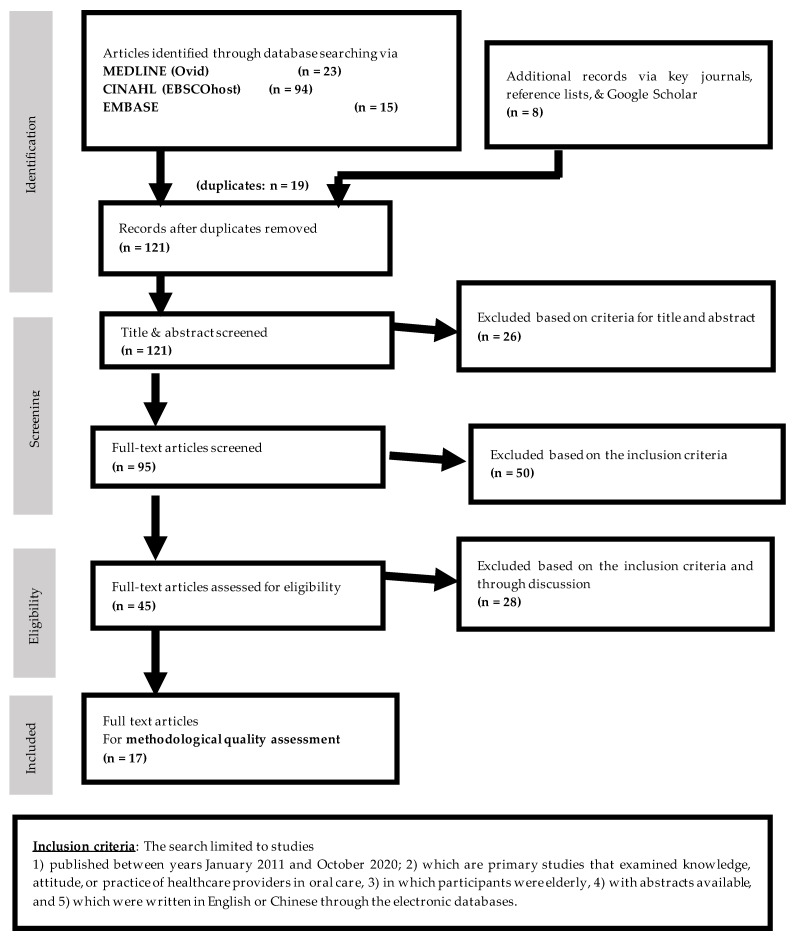
Flow of searching and inclusion of relevant articles.

Literature review was conducted through MEDLINE (OvidSP), EMBASE, and CINAHL databases. After a comprehensive selection by screening titles and abstracts, and hand-searching as well as removal of duplicates, a total of 121 articles were obtained. Abstract of each article was then reviewed. There were 26 articles excluded based on the criteria for title and abstract. A total of 95 articles were obtained for further reviewed. Then, there were 50 articles excluded based on inclusion criteria. Therefore, 45 articles were assessed for eligibility. In this stage, articles were excluded based on the inclusion criteria and through discussion. At last, a total of 17 articles (1 qualitative study and 16 quantitative studies) were selected to further review for content and domain specification and item generation. Figure 2 illustrates the flow of the searching and inclusion of relevant articles.

The tool was developed through selecting appropriate items with reference to the questionnaires of relevant articles. The COM-B model was used to guide the domain identification. During this process, the domain named as ‘practice’ instead of ‘behavior’ was determined based on appropriateness and relevancy of understanding healthcare providers’ practice in oral care of institutionalized elderly residents. In addition, the items included in ‘practice’ were more appropriate to reflect the practice-related issues, such as item #7, ‘I always perform oral care according to the instruction of my unit head or the protocol at my workplace’. 

At last, three domains, knowledge (21 items), attitude (15 items), and practice (15 items) were selected in the preliminary version. Knowledge was rated by ‘Yes’, ‘No’, and ‘Don’t know’. Attitude and practice were rated using 5-likert scales. The preliminary version of the KAP tool was sent to the experts for review and CVI.

### 3.2. Expert Review in Two Stages and CVI Results

The drafts in English and Chinese versions were reviewed by three experts in the first stage. Modifications were made according to their comments via email or telephone. The items were selected from the relevant articles and redesigned to suit the Chinese culture and current local healthcare dental services. For example, item #13 in knowledge part, ‘It is normal to lose teeth as one gets old’ reflected the misconception about loss of teeth is a normal progress if getting old in Chinese culture. Another item #5 in the practice part, ‘I use interdental brush to clean adjacent tooth surfaces for residents with large space between teeth’ is to understand if a healthcare provider will perform dental care using appropriate equipment as using interdental brush is not a usual practice in the Chinese population. Moreover, item #13 in the attitude part, ‘The outreach dentist programme is helpful in assisting us to deliver oral care to the residents’ is to understand how the healthcare providers perceive this new and important healthcare dental service supported by the government specifically to institutionalized elderly people. A total of six items were deleted due to duplication of meaning or being not essential. Four items were moved to more appropriate domains. The second version of the KAP tool consists of 19 items in knowledge, 13 items in attitude, and 11 items in practice.

At the second stage, another three experts (one community dentist, one dentist psychologist, and one dentist in faculty of dentistry of a university) were invited to evaluate the tool independently [18]. They evaluated the domains and items for their appropriateness, structure and clarity, redundant inquiries, and ambiguity in meaning. All items of the preliminary version were reviewed and commented by these three experts rating from 2 to 4 for CVI. Modification or elimination was made if narrative comments were given. 

The CVI and CVR were calculated to evaluate content validity [25,26,27]. The I-CVI calculations for the relevancy of each item. All of forty-three items (100%) were rated relevant (score 3 or 4) and the I-CVI was 1.00. Besides, the universal agreement was calculated by the sum of all I-CVI which was 43 and then divided by 43. The result of the S-CVI/UA was 1.00, demonstrating excellent content validity. The expert review result is illustrated in Table 1. The CVI and CVR results are shown in Table 2.

### 3.3. Tool Refinement

There were two rounds of evaluations by the Delphi panel [26]. Items were rephrased for clarity, moved to a more suitable domain, or deleted if duplicated or not essential in the first round. A total of six duplicated or not essential items were deleted, and one item was added. After the CVI and cognitive interviews, all items were evaluated and included. The final version of the tool consists of three domains (KAP) and 43 items (Table S1). The applicability and feasibility of the tool was examined using a pilot study. The subjects were also asked if they confronted difficulties in understanding and answering the items. 

### 3.4. A Pilot Study

A total of 20 subjects, who were healthcare providers in the LTC institution, were recruited in the pilot study. There were 11 male subjects (55%). Mean age was 33.6 (SD 10.53) years old. All healthcare providers had attained secondary and tertiary levels of education. Most of them (*n* = 14, 70%) were working at high-care level LTC institutions. About 65% (*n* = 13) were nurses. More than half of them (*n* = 12, 60%) had at least two-years working experience in LTC services. Almost 75% of them needed to take care of at least more than 20 elderly residents in a shift. Apart from nurses, only 20% (*n* = 4) other healthcare providers had received oral care training. Among 20 healthcare providers, 25% had not performed oral care for elderly residents. Most of them (65%) performed oral care for their elderly residents at least twice a day. About 70% of them had at least one elderly resident who needs oral care in a shift. However, only a few (*n* = 5) reported that oral care was the first priority in their daily practice. About 60% of healthcare providers knew that there was a guideline for oral care in their workplace. 

The scoring of knowledge domain was using ‘Yes’, ‘No’, and ‘Don’t know’. Only correct answers were scored ‘2’ but incorrect or ‘Don’t know’ answers were scored ‘1’. Some items of attitudes were reverse items that rated “1 = very agree” and “5 = very disagree”. All reverse items were determined when negative response to oral care was present, for example, ‘Oral care is an unpleasant task’ or the inappropriate attitude was present, for example, ‘I will only perform oral care to residents who are willing to open their mouth’. All reverse items were asterisked, and they were handled before data analysis. The test-retest reliability scores for internal consistency of knowledge, attitude, practice, and overall KAP were 0.67, 0.93, 0.92, and 0.94, respectively.

## 4. Discussion

The present study was conducted mainly on content validity and reliability analyses to ensure that the items in each dimension (knowledge, attitude, and practice) were rightly placed. The items were structured based on the literature review with relevant papers and expertise of respective professionals in dentistry. Moreover, most of subjects were institutionalized healthcare providers. As a result, the validity and reliability were enhanced.

The KAP tool provides a unique self-report assessment to understand KAP of institutionalized healthcare providers in oral care of elderly people. Since institutionalized elderly people are commonly weaker and more self-care dependent, their oral care relies on healthcare providers. Although poor oral care of institutionalized elderly people can be due to numerous factors, KAP of healthcare providers in oral care of elderly people should be examined. Therefore, the validated tool in this study is important to evaluate the KAP of healthcare providers and the results can direct appropriate strategies to improve KAP of healthcare providers. 

According to past studies, institutionalized healthcare providers had inadequate knowledge about oral health and oral care of elderly people [15,28,29]. Strategies to increase the knowledge of oral health and oral care can help foster the understanding and importance of oral health attributed to appropriate oral care performed by healthcare providers. Knowledge enhancement increases self-confidence and improves attitude and practice in oral care. Therefore, the validated tool is valuable to understanding KAP of healthcare providers in order to develop appropriate strategies to provide support to healthcare providers for promoting better oral health of institutionalized elderly people.

The literature review, together with the use of the COM-B model for determining the domains and designing the structure of the KAP tool, is essential. The literature review provides rigorous approaches to select and develop appropriate items in each domain guided by the COM-B model. Some common items can be retrieved or referenced based on the relevant studies. To ensure applicability of the tool in institutionalized healthcare providers, three experts were invited for structuring and designing the initial tool and another five experts in evaluating each item of the tool and giving comments on each item in the respective domain, and five experts to conduct content validity. All experts were specialized in clinical practice or teaching at dentistry. Content validity measures how well the designed items reflect specific domains through assessing I-CVI, S-CVI, and S-CVI/UA [26], which were all indicated to be relevant and excellent. 

Further clarification and rephrasing were the primary reasons for modification of the tool. Multiple reviews by the experts were crucial in tool development through cognitive assessments and iterative revisions [21]. The final version of the tool has been reached by compromise between the experts. Input given by experts in respective field increases the accuracy and applicability of the tool in the process of content validity. Qualitative approach was conducted to collect more valuable information from institutionalized healthcare providers also for content validity [21]. This approach understands how well the healthcare providers answered the survey questions and identify potential problems that cause response errors. Their feedback on the overall format of the tool was obtained to improve the applicability and feasibility [21,26]. 

Overall, a rigorous literature review, multiple recommendations/reviews by experts in the relevant field, and narratives provided by the institutionalized healthcare providers are essential for the tool development [26]. The development process through literature review, guided by the health model, and with advice from experts in multiple stages, increases accuracy and applicability of the tool. The quantitative analysis of the content validity of items was excellent. Further feedback from respective target group enhances accuracy and applicability of the tool [21,25,26]. The pilot study has played a vital role in confirming the accuracy of items and testing the applicability and feasibility. The test-retest reliability results indicated acceptable to excellent. The length of the tool is appropriate which takes about 20 to 30 min to be completed. 

Based on the demographic and clinical results, most of the healthcare providers were expected to be responsible for taking care of at least 20 elderly residents and they needed to perform oral care to at least two elderly residents at least twice a day in a shift. Only 25% (*n* = 5) would put oral care as the first priority. According to this high ratio of staff and elderly resident number and lower priority, oral care of elderly residents, particularly those who are more self-care dependent, may be neglected. Additionally, only a few healthcare providers received oral care training. This increases the vulnerability of oral health in elderly residents. Therefore, understanding KAP of healthcare providers in oral care of elderly residents is important to developing strategies to improve oral care practice of healthcare providers. Subsequently, oral health of elderly residents can be maintained. 

The tool has been developed to measure KAP of healthcare providers in oral care of institutionalized elderly people, and can be potentially used in research and for practice purposes. Researchers can use this tool to understand KAP of healthcare providers in oral care of institutionalized elderly people in order to devise strategies to improve KAP of this specific healthcare group. This tool can be also used to evaluate KAP of healthcare providers who are newly employed so that appropriate content will be included in staff training. 

### Strengths and Weaknesses

The present study developed and validated a tool to evaluate KAP of healthcare providers in oral care of institutionalized elderly people. However, this tool may not be generalizable to other populations. The items are designed to fit for the research in the target population in institutionalized healthcare providers for their KAP on oral care of elderly people. Therefore, the tool may need to be modified and validated before applied in another population, for example, healthcare professionals in clinical settings. Due to the small sample size, further factor analysis is infeasible (Kaiser–Meyer–Olkin measure of sampling adequacy <0.5). A large sample is needed for further validation of the tool in future studies. 

## 5. Conclusions

The tool is the first instrument to assess KAP of healthcare providers in oral care of elderly people in LTC institutions. The development of the KAP tool used a mixed-method approach to design and structure items relevant to KAP of institutionalized healthcare providers in oral care of elderly residents. The tool has presented high content validity and reliability. This tool can benefit in both research and practice purposes for suggesting strategies, such as staff education in oral care of institutionalized elderly people.

## Figures and Tables

**Table 1:** Expert review for relevancy check and comments.

No.	Item Questions	Interpretation	Recommendations
KQ1	Oral health is directly related to general health.	Relevant	Included
KQ2	Fluorides can help protect dental health.	Relevant	Included
KQ3	Toothbrushing should be done in the morning after waking up and before bed at night every day.	Relevant	Rephrased
KQ4	Sugary food, e.g., candy, increases the risk of tooth decay in residents.	Relevant	Included
KQ5	Dental plaque can cause gum diseases and dental caries.	Relevant	Rephrased
KQ6	Medication is one of the common reasons for dry mouth.	Relevant	Included
KQ7	Dry mouth increases the risk of oral problems.	Relevant	Rephrased
KQ8	Interdental cleaning aids, such as dental floss and interdental brush, can be used to clean the adjacent tooth surfaces.	Relevant	Included
KQ9	Mouth rinsing can replace toothbrushing.	Relevant	Included
KQ10	It is normal that the residents feel toothache and sores in their mouth.	Relevant	Included
KQ11	Denture can totally replace natural teeth.	Relevant	Included
KQ12	Denture should be taken out at night, cleaned and soaked.	Relevant	Included
KQ13	It is normal to lose teeth as one gets old.	Relevant	Rephrased
KQ14	Residents with full denture do not need to see a dentist.	Duplicatedwith KQ19	Deleted
KQ15	Unfit denture may indicate serious oral problems.	Relevant	Rephrased
KQ16	Annual dental check is as important as body check.	Relevant	Included
KQ17	Dental plaque does not form on denture.	Relevant	Rephrased
KQ18	Residents with denture need to have regular dental check.	Duplicated with KQ 19	Deleted
KQ19	Residents with no teeth need to have dental check regularly.	Relevant	Included
KQ20	The residents with full denture only need mouth rinsing.	Relevant	Included
KQ21	Residents with tubing for feeding need oral care.	Relevant	Added
AQ1	Daily oral care is an essential procedure.	Relevant	Rephrased
AQ2	I will perform oral care for residents at least once in my shift.	Relevant	Moved to P (PQ15)
AQ3	Independent residents should clean their dentures by themselves.	Relevant	Rephrased
AQ4	When I am busy, I tend to ignore oral care to residents.	Relevant	Included
AQ5	Oral care is an unpleasant task.	Relevant	Included
AQ6	Oral care training for residents can improve my practice skills.	Relevant	Included
AQ7	Oral care is not dentist’s duty.	Not essential	Deleted
AQ8	I would perform other care procedures instead of oral care procedures to the residents.	Relevant	Rephrased and moved from P (PQ14)
AQ9	It is normal if the gum bleeds while doing oral care to a resident. There is usually no need to follow up.	Relevant	Rephrased
AQ10	I will only perform oral care to residents who are willing to open their mouth.	Relevant	Rephrased
AQ11	Oral care to residents is my duty.	Relevant	Included
AQ12	I will assist residents to perform oral care only if they have difficulty.	Relevant	Included
AQ13	I am willing to spend time on oral care for each resident.	Relevant	Rephrased
AQ14	If a resident requests to see a dentist, I am responsible to arrange for making corresponding arrangements.	Relevant	Included
AQ15	The outreach dentist programme is helpful in assisting us to deliver oral care to the residents.	Relevant	Added
PQ1	I will assist all residents in their toothbrushing or wiping their mouths at least once in my shift.	Relevant	Rephrased
PQ2	I will not give junk foods to residents.	Not essential	Deleted
PQ3	I will position the resident for oral care.	Not essential	Deleted
PQ4	While I am performing oral care, I will brush the resident’s teeth.	Relevant	Rephrased
PQ5	While I am performing oral care, I will brush the margin between teeth and gum.	Relevant	Included
PQ6	While I am performing oral care, I will brush the resident’s tongue.	Relevant	Rephrased
PQ7	I use interdental brush to clean adjacent tooth surfaces for residents with large space between teeth.	Relevant	Included
PQ8	I will not perform oral care to residents if there is a risk of choking during the procedure.	Relevant	Rephrased
PQ9	I always perform oral care according to the instruction of my unit head or the protocol at my workplace.	Relevant	Included
PQ10	While performing oral care, I will do oral assessment for the resident.	Relevant	Included
PQ11	I will refer residents with oral problems to a dentist.	Relevant	Included
PQ12	I will inform my senior when I have found oral problems in the resident.	Relevant	Included
PQ13	I can find adequate equipment to perform oral care to residents in my workplace.	Relevant	Included
PQ14	I would perform other care procedures instead of oral care procedures to the residents.	Relevant	Moved from A (AQ8)
PQ15	I will perform oral care for residents at least once in my shift.	Not essential	Moved from A (AQ2)but deleted

NOTE: Number of items considered relevant and included by all experts, *n* = 3.

**Table 2:** Calculation of the I-CVI and CVR for each item.

No.	Item Questions	I-CVI	Interpretation	Recommendations	CVR	Interpretation
KQ1	Oral health is directly related to general health.	1.00	Relevant	Included	1.00	Included
KQ2	Fluorides can help protect dental health.	1.00	Relevant	Included	1.00	Included
KQ3	Toothbrushing should be done in the morning after waking up and before bed at night every day.	1.00	Relevant	Included	1.00	Included
KQ4	Sugary food, e.g., candy, increases the risk of tooth decay in residents.	1.00	Relevant	Rephrased	1.00	Included
KQ5	Dental plaque can cause gum diseases and dental caries.	1.00	Relevant	Included	1.00	Included
KQ6	Medication is one of the common reasons for dry mouth.	1.00	Relevant	Included	1.00	Included
KQ7	Dry mouth increases the risk of oral problems.	1.00	Relevant	Rephrased	1.00	Included
KQ8	Interdental cleaning aids, such as dental floss and interdental brush, can be used to clean the adjacent tooth surfaces.	1.00	Relevant	Included	1.00	Included
KQ9	Mouth rinsing can replace toothbrushing.	1.00	Relevant	Rephrased	1.00	Included
KQ10	It is normal that the residents feel toothache and sores in their mouth.	1.00	Relevant	Included	1.00	Included
KQ11	Denture can totally replace natural teeth.	1.00	Relevant	Included	1.00	Included
KQ12	Denture should be taken out at night, cleaned and soaked.	1.00	Relevant	Included	1.00	Included
KQ13	It is normal to lose teeth as one gets old.	1.00	Relevant	Included	1.00	Included
KQ15	Unfit denture may indicate serious oral problems.	1.00	Relevant	Included	1.00	Included
KQ16	Annual dental check is as important as body check.	1.00	Relevant	Included	1.00	Included
KQ17	Dental plaque does not form on denture.	1.00	Relevant	Included	1.00	Included
KQ19	Residents with no teeth need to have dental check regularly.	1.00	Relevant	Included	1.00	Included
KQ20	The residents with full denture only need mouth rinsing.	1.00	Relevant	Included	1.00	Included
KQ21	Residents with tubing for feeding need oral care.	1.00	Relevant	Included	1.00	Included
AQ1	Daily oral care is an essential procedure.	1.00	Relevant	Added	1.00	Included
AQ3	Independent residents should clean their dentures by themselves.	1.00	Relevant	Rephrased	1.00	Included
AQ4	When I am busy, I tend to ignore oral care to residents.	1.00	Relevant	Included	1.00	Included
AQ5	Oral care is an unpleasant task.	1.00	Relevant	Included	1.00	Included
AQ7	Oral care training for residents can improve my practice skills.	1.00	Relevant	Included	1.00	Included
AQ8	I would perform other care procedures instead of oral care procedures to the residents.	1.00	Relevant	Included	1.00	Included
AQ9	It is normal if the gum bleeds while doing oral care to a resident. There is usually no need to follow up.	1.00	Relevant	Included	1.00	Included
AQ10	I will only perform oral care to residents who are willing to open their mouth.	1.00	Relevant	Included	1.00	Included
AQ11	Oral care to residents is my duty.	1.00	Relevant	Rephrased	1.00	Included
AQ12	I will assist residents to perform oral care only if they have difficulty.	1.00	Relevant	Included	1.00	Included
AQ13	I am willing to spend time on oral care for each resident.	1.00	Relevant	Added	1.00	Included
AQ14	If a resident requests to see a dentist, I am responsible to arrange for making corresponding arrangements.	1.00	Relevant	Included	1.00	Included
AQ15	The outreach dentist programme is helpful in assisting us to deliver oral care to the residents.	1.00	Relevant	Included	1.00	Included
PQ1	I will assist all residents in their toothbrushing or wiping their mouths at least once in my shift.	1.00	Relevant	Included	1.00	Included
PQ4	While I am performing oral care, I will brush the resident’s teeth.	1.00	Relevant	Included	1.00	Included
PQ5	While I am performing oral care, I will brush the margin between teeth and gum.	1.00	Relevant	Included	1.00	Included
PQ6	While I am performing oral care, I will brush the resident’s tongue.	0.80	Relevant	Included	0.71	Included
PQ7	I use interdental brush to clean adjacent tooth surfaces for residents with large space between teeth.	1.00	Relevant	Included	1.00	Included
PQ8	I will not perform oral care to residents if there is a risk of choking during the procedure.	1.00	Relevant	Included	1.00	Included
PQ9	I always perform oral care according to the instruction of my unit head or the protocol at my workplace.	1.00	Relevant	Included	1.00	Included
PQ10	While performing oral care, I will do oral assessment for the resident.	1.00	Relevant	Included	1.00	Included
PQ11	I will refer residents with oral problems to a dentist.	0.80	Relevant	Included	0.71	Included
PQ12	I will inform my senior when I have found oral problems in the resident.	1.00	Relevant	Included	1.00	Included
PQ13	I can find adequate equipment to perform oral care to residents in my workplace.	1.00	Relevant	Included	1.00	Included

NOTE: Number of experts evaluated the item essential. CVR = (Ne − N/2)/(N/2) with 5 experts (*n* = 5), items with the CVR bigger than 0.99 remained in the questionnaire.

## Data Availability

The data presented in this study are available on request from the corresponding author. The data are not publicly available due to privacy reason.

## Supplementary Materials

The following are available online at https://www.mdpi.com/article/10.3390/ijerph18084145/s1. Table S1: The knowledge, attitudes, and practices questionnaire.

## References

[1] World Health Organization (WHO) (2020). Oral Health. https://www.who.int/news-room/fact-sheets/detail/oral-health.

[2] Gao S.S., Chen K.J., Duangthip D., Lo E.C.M., Chu C.H. (2018). Oral health care in Hong Kong. Healthcare.

[3] Hopcraft M.S., Morgan M.V., Satur J.G., Wright C., Darby I.B. (2012). Oral hygiene and periodontal disease in Victorian nursing homes. Gerodontology.

[4] Li X.L., Liu M.Y., Cheng L., Zhu H.F., Shang S.H., Cui D. (2018). Impact of comprehensive health education on oral care knowledge, attitude and practice in the elderly in long-term care institutions. SJS.

[5] Lin H.C., Wong M.C.M., Wang Z.J., Lo E.C.M. (2001). Oral health knowledge, attitudes, and practices of Chinese Adults. J. Dent. Res..

[6] Chalmers J., Pearson A. (2005). Oral hygiene care for residents with dementia: A literature review. JAN.

[7] Zhu L., Petersen P.E., Wang H.Y., Bian J.Y., Zhang B.X. (2005). Oral Health Knowledge, Attitudes and Behaviour of Adults in China. Int. Dent. J..

[8] Zuluaga D.J.M., Ferreira J., Montoya J.A., Willumsen T. (2012). Oral health in institutionalised elderly people in Oslo, Norway and its relationship with dependence and cognitive impairment. Gerodontology.

[9] Barrios R., Tsakos G., Garcia-Medina B., Martinez-Lara I., Bravo M. (2014). Oral health-related quality of life and malnutrition in patients treated for oral cancer. Support. Care Cancer.

[10] Dharamsi S., Jivani K., Dean C., Wyatt C. (2009). Oral care for frail elders: Knowledge, attitudes, and practices of long-term care staff. J. Dent. Educ..

[11] Lo E.C., Luo Y., Dyson J.E. (2004). Oral health status of institutionised elderly in Hong Kong. Community Dent. Health.

[12] Wiener R.C., Meckstroth R. (2014). The Oral Health Self-Care Behavior and Dental Attitudes among Nursing Home Personnel. J. Stud. Soc. Sci..

[13] Porter J., Ntouva A., Read A., Murdoch M., Ola D., Tsakos G. (2015). The impact of oral health on the quality of life of nursing home residents. Health Qual. Life Outcomes.

[14] Atchison K.A., Dolan T.A. (1990). Development of the geriatric oral health assessment index. J. Dent. Educ..

[15] Maille G., Saliba-Serre B., Ferrandez A.M., Ruquet M. (2017). Use of care and the oral health status of people aged 60 years and older in France: Results from the National Health and Disa-bility Survey. Clin. Interv. Aging.

[16] Wong M.F.F., Ng T.Y.Y., Leung W.K. (2019). Oral health and its associated factors among older institutionalized residents—A systematic review. IJERPH.

[17] Arpin S., Brodeur J.M., Corbeil P. (2008). Dental caries, problems perceived and use of services among institutionalized elderly in 3 regions of Quebec, Canada. JCDA.

[18] Hearn L., Slack-Smith L. (2014). Oral health care in residential aged care services: Barriers to engaging health-care providers. Aust. J. Prim. Health.

[19] Stančić I., Petrović M., Popovac A., Vasović M., Despotović N. (2016). Caregivers’ attitudes, knowledge and practices of oral care at nursing homes in Serbia. Vojnosanit. Pregl..

[20] Vasudevan V., Rimmer J.H., Kviz F. (2015). Development of the barriers to physical activity questionnaire for people with mobility impairments. Disabil. Health J..

[21] Collins D. (2003). Pretest survey instruments: An overview of cognitive methods. Qual. Life Res..

[22] Paryag A., Rafeek R., Lewis D. (2016). Knowledge, Attitudes, Beliefs and Training of Care Givers and Nursing Staff in Relation to Oral Care in Institutions for Older People in Trinidad. Int. J. Dent. Oral. Health.

[23] Sinavarat P., Manosoontorn S., Anunmana C. (2018). Knowledge, attitudes, and behavior towards oral health among a group of staff caring for elderly people in long-term care facilities in Bangkok, Thailand. M Dent. J..

[24] Michie S., van Stralen M.M., West R. (2011). The behaviour change wheel: A new method for characterizing and designing behavior change interventions. Implement. Sci..

[25] Armstrong T.S., Cohen M.Z., Eriksen L., Cleeland C. (2005). Content validity of selfreport measurement instruments: An illustration from the development of the brain tumor module of the M.D. Anderson symptom inventory. Oncol. Nurs. Forum..

[26] Zamanzadeh V., Ghahramanian A., Rassouli M., Abbaszadeh A., Alavi H. (2015). Design and implementation content validity Study: Development of an instrument for measuring patient-centered communication. J. Caring Sci..

[27] Yamada J., Stevens B., Sidani S., Watt-Watson J., De Silva N. (2010). Content validity of a process evaluation checklist to measure intervention implementation fidelity of the EPIC intervention. Worldviews Evid. Based Nurs..

[28] Khanagar S., Naganandini S., Tuteja J.S., Naik S., Satish G., Divya K.T. (2015). Improving oral hygiene in institutionalized elderly by educating their caretakers in Bangalore city, India: A randomized control trial. Can. Geriatr. J..

[29] Unfer B., Braun K.O., De Oliveira Ferreira A.C., Ruat G.R., Batista A.K. (2012). Challenges and barriers to quality oral care as perceived by caregivers in long-stay institutions in Brazil. Gerodontology.

