# Supplementary material for: First Data in the Process of Validating a Tool to Evaluate Knowledge, Attitude, and Practice of Healthcare Providers in Oral Care of Institutionalized Elderly Residents: Content Validity, Reliability and Pilot Study
Source: Int J Environ Res Public Health. 2021 Apr 14;18(8):4145. doi: 10.3390/ijerph18084145 (PMC8070937; doi:10.3390/ijerph18084145)
Supplement: Supplementary file 1 [file ijerph-18-04145-s001.pdf]

Table S1

## Survey of knowledge, attitude, and practice of healthcare workers in oral care of elderly of long-term care institutions

| I. | Knowledge<br>According to my understanding, … | Yes | No | Don't know |
|---|---|---|---|---|
| 1 | Oral health is directly related to general health. | | | |
| 2 | Fluorides can help protect dental health. | | | |
| 3 | Toothbrushing should be done in the morning after waking up and before bed at night every day. | | | |
| 4 | Sugary food, eg candy, increases the risk of tooth decay in residents. | | | |
| 5 | Dental plaque can cause gum diseases and dental caries. | | | |
| 6 | Medication is one of the common reasons for dry mouth. | | | |
| 7 | Dry mouth increases the risk of oral problems. | | | |
| 8 | Interdental cleaning aids, such as dental floss and interdental brush, can be used to clean the adjacent tooth surfaces. | | | |
| 9 | Mouth rinsing can replace toothbrushing. | | | |
| 10 | It is normal that the residents feel toothache and sores in their mouth. | | | |
| 11 | Denture can totally replace natural teeth. | | | |
| 12 | Denture should be taken out at night, cleaned and soaked. | | | |
| 13 | It is normal to lose teeth as one gets old. | | | |
| 14 | Unfit denture may indicate serious oral problems. | | | |
| 15 | Annual dental check is as important as body check. | | | |
| 16 | Dental plaque does not form on denture. | | | |
| 17 | Residents with no teeth need to have dental check regularly. | | | |
| 18 | The residents with full denture only need mouth rinsing. | | | |
| 19 | Residents with tubing for feeding need oral care. | | | |

| II. | Attitude<br>Upon my opinion, I think… | Strongly disagree → Strongly agree | | | | |
|---|---|---|---|---|---|---|
| | | 1 | 2 | 3 | 4 | 5 |
| 1 | Daily oral care is an essential procedure. | | | | | |
| 2 | Independent residents should clean their dentures by themselves. | | | | | |
| 3* | When I am busy, I tend to ignore oral care to residents. | | | | | |
| 4* | Oral care is an unpleasant task. | | | | | |
| 5 | Oral care training for residents can improve my practice skills. | | | | | |
| 6* | I would perform other care procedures instead of oral care procedures to the residents. | | | | | |
| 7* | It is normal if the gum bleeds while doing oral care to a resident. There is usually no need to follow up. | | | | | |
| 8* | I will only perform oral care to residents who are willing to open their mouth. | | | | | |
| 9 | Oral care to residents is my duty. | | | | | |
| 10 | I will assist residents to perform care if they have difficulty. | | | | | |
| 11* | I am willing to spend time on oral care for each resident. | | | | | |
| 12 | If a resident requests to see a dentist, I am responsible to arrange for making corresponding arrangements. | | | | | |
| 13 | The outreach dentist programme is helpful in assisting us to deliver oral care to the residents. | | | | | |

| **III.** Practice<br>In the past 3 months, … | | Strongly disagree → Strongly agree | | | | |
|---|---|---|---|---|---|---|
| | | 1 | 2 | 3 | 4 | 5 |
| 1 | **I will assist all residents in their toothbrushing or wiping their mouths at least once in my shift.** | | | | | |
| 2 | **While I am performing oral care, I will brush the resident's teeth.** | | | | | |
| 3 | **While I am performing oral care, I will brush the margin between teeth and gum.** | | | | | |
| 4 | **While I am performing oral care, I will brush the resident's tongue.** | | | | | |
| 5 | **I use interdental brush to clean adjacent tooth surfaces for residents with large space between teeth.** | | | | | |
| 6 | **I will not perform oral care to residents if there is a risk of choking during the procedure.** | | | | | |
| 7 | **I always perform oral care according to the instruction of my unit head or the protocol at my workplace.** | | | | | |
| 8 | **While performing oral care, I will do oral assessment for the resident.** | | | | | |
| 9 | **I will refer residents with oral problems to a dentist.** | | | | | |
| 10 | **I will inform my senior when I have found oral problems in the resident.** | | | | | |
| 11 | **I can find adequate equipment to perform oral care to residents in my workplace.** | | | | | |

Thank you very much!
